# Relative position of cerclage – an ultrasound marker for prediction of preterm birth

**DOI:** 10.1186/s12884-026-08872-1

**Published:** 2026-02-26

**Authors:** Laura van der Krogt, Lisa Story, Andrew Shennan, Natalie Suff

**Affiliations:** Department of Women and Children’s Health, King’s College London, St Thomas’ Hospital, 10th Floor, North Wing, SE1 7UH 020 7188 3639 London, United Kingdom

**Keywords:** Spontaneous preterm birth, Cervical cerclage, Cervical length, Ultrasound

## Abstract

**Background:**

A transvaginal cerclage (TVC) identified high in the cervix is associated with prolongation of pregnancy. However, the relative position of the TVC (length above the cerclage/total cervical length) may be a better predictor of cerclage success as it is proportional of total cervical length. This study evaluates the relative position of TVC and its association with spontaneous preterm birth, comparing its predictive value against other post-cerclage ultrasound parameters.

**Methods:**

A retrospective cohort study was conducted of singleton pregnancies with TVC. Demographic and ultrasound data were collected. Differences in TVC characteristics between those who delivered preterm (< 37 weeks) and term, including relative position of cerclage, were assessed using Student’s t-test where data was continuous and chi-squared test where categorical. Receiver operator curves were generated to evaluate predictive performance (SPSS version 29.0).

**Results:**

Among 53 women, 25% (*n* = 13) delivered preterm. The relative position of TVC was significantly lower in preterm cases (mean 0.41 versus 0.52, *p* = 0.024), while total cervical length showed no difference. Length above the TVC was shorter in spontaneous preterm births (mean 11 mm versus 16 mm; *p* = 0.008). In those who delivered preterm, findings of funnelling and amniotic fluid sludge were more likely to be present after cerclage (*p* = 0.07 and *p* = 0.02 respectively). ROC analysis demonstrated good predictive ability for relative position (AUC 0.74) and length above the TVC (AUC 0.73), with an optimal threshold of 0.425 for relative position. Cervical length and cerclage height were poor predictors (AUC 0.43 and 0.59 respectively).

**Conclusion:**

Ultrasound assessment after cerclage can identify women at higher risk of spontaneous preterm birth. Relative position and length above the TVC are good predictors, whereas cervical length and cerclage height are not. Incorporating relative position of TVC position into clinical risk stratification warrants further evaluation.

## Background

Preterm birth, where delivery occurs prior to 37 weeks’ completed gestation, remains an important global issue with approximately 1 in 10 infants being born prematurely worldwide [1]. Globally it is the leading cause of neonatal morbidity and mortality [1]. The aetiology of spontaneous preterm birth (sPTB) is multifactorial, ending in a final pathway of cervical softening, shortening, dilatation and delivery [2].

Transvaginal cervical cerclage (TVC), a stitch placed around the cervix, is a surgical intervention for the prevention of mid-trimester loss and PTB [3]. A TVC may be indicated based on risk factors (history-indicated), ultrasound findings of a short cervix < 25 mm (ultrasound-indicated) or when the cervix has dilated with membranes exposed (emergency) [3]. According to a Cochrane review, pregnant women at high risk of preterm birth with TVC were less likely to have preterm births before 28, 34 and 37 weeks completed gestation, compared to controls without TVC (average RR 0.77, 95% CI 0.66 to 0.89) [4]. Although the mechanism by which TVCs prevent preterm delivery is unknown, they may provide mechanical support and/or act as a barrier to ascending infection by maintaining cervical length and the endocervical mucus plug [5].

Preterm deliveries can occur despite insertion of TVC, with rates varying between 20 and 70% depending on the indication for the cerclage [6]. Previous studies indicate that post-cerclage cervical length and longitudinal cervical length measurements are a good predictor of preterm delivery in women following cerclage insertion [7, 8]. It has also been reported that a TVC placed high in the cervix is associated with a decreased risk [9–12]. However, the height of TVC does not account for the total length of the cervix. Moreover, it is difficult to delineate whether the cerclage is high in a stable cervix or there is dynamic change above the stitch. Therefore, its relative position (the length above the TVC as a proportion of the total cervical length) may provide an additional value in prediction and risk stratification. In addition, other parameters such as funnelling and sludge after cerclage may aide in determining the likelihood of subsequent preterm delivery [13–16]. This study therefore aims to assess relative position of the TVC in addition to other ultrasound parameters to determine if these factors are related to mid-trimester loss and sPTB after TVC insertion.

## Methods

A retrospective cohort study of women who received a TVC at a tertiary-level London hospital and had consented to inclusion in the Preterm Clinical Network Database from 2016 to 2021 was performed (research ethics committee reference number: 16/ES/0093). Women were included if the TVC was performed prior to 24 weeks’ gestation in a singleton pregnancy; those who had a repeat TVC, iatrogenic preterm delivery or no recorded pregnancy outcome were excluded.

TVC was performed when indicated based on history (previous cerclage, previous mid-trimester loss, or previous preterm birth), ultrasound findings (cervical length < 25 mm), or visible membranes requiring rescue cerclage. The surgical approach (McDonald or Shirodkar) was determined by the operating surgeon. After cerclage placement, patients attended the preterm birth clinic for cervical length surveillance. Scans were performed by trained staff using standardized transvaginal techniques, including correct landmark identification, image optimisation, three reproducible measurements, and assessment of dynamic changes with fundal pressure.

Maternal demographic data, indication for TVC (history-indicated, ultrasound-indicated and rescue cerclage), gestation of TVC insertion, type of TVC (McDonald and Shirodkar) and gestation at delivery were collected. Ultrasound parameters collected from transvaginal ultrasound findings included cervical length before and after TVC insertion, length above the TVC (measured as a straight line from TVC to internal os), cerclage height (measured as a straight line from the external os to TVC), presence of funnelling and amniotic fluid sludge.

**Fig. 1:**
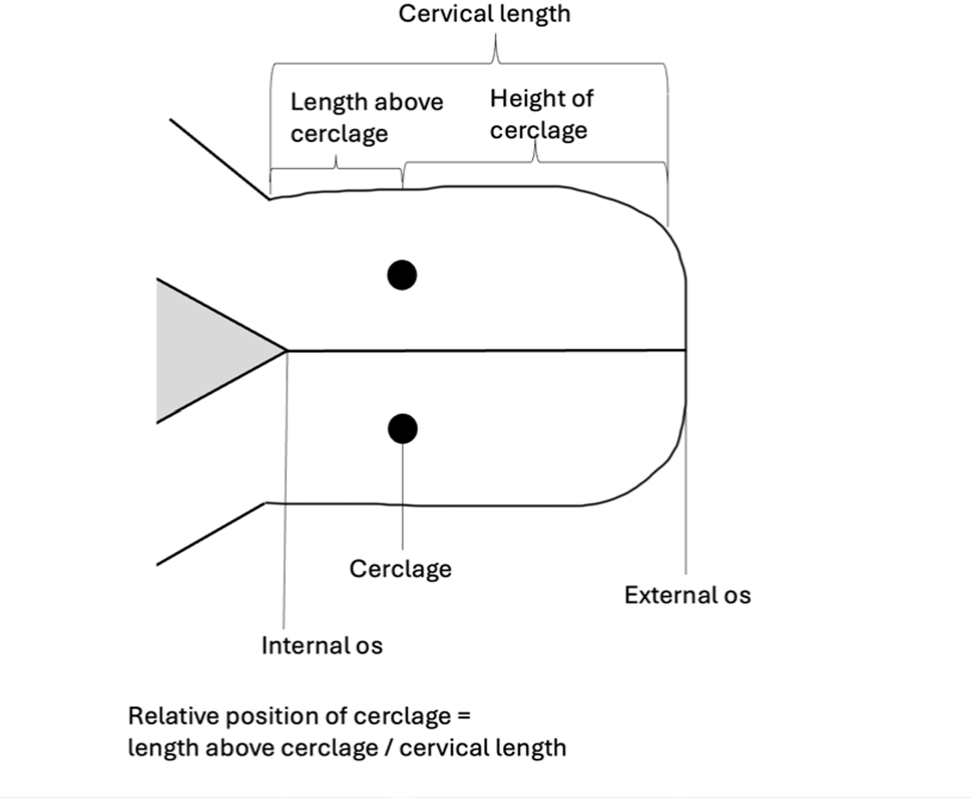
Diagram of cervical measures used in this study

The relative position of the TVC was determined by creating a ratio of the length of cervix above the TVC as a proportion of the total cervical length following the procedure, see Fig. 1.

Differences in TVC characteristics between women who subsequently delivered at term (at or greater than 37 weeks’ gestation) and those that delivered preterm (before 37 weeks’ gestation) were analysed by student’s t-test where data was continuous and chi-squared where data was categorical. The performance of the different post-cerclage ultrasound measurements for predicting sPTB was assessed using Receiver Operating Characteristic (ROC) curves and an optimum threshold for relative position of TVC identified (SPSS version 29.0).

## Results

**Fig. 2:**
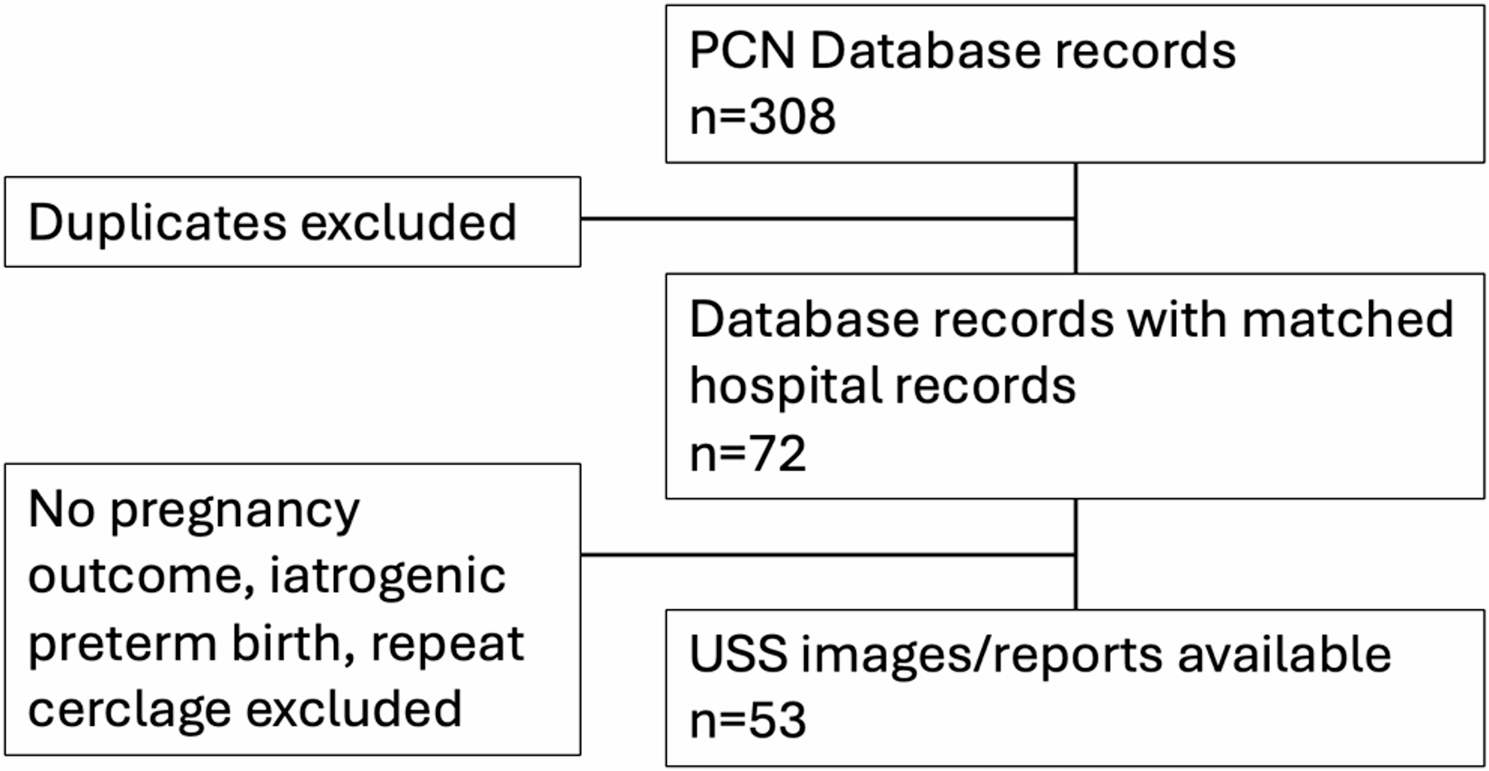
Total records reviewed, and number included in analysis

Fifty-three cases were suitable for analysis. Of these 13 (25%) delivered preterm and 40 (75%) delivered at term. Exclusions can be seen in Fig. 2.

Maternal demographics including age, body mass index, ethnicity, gravidity and parity were similar across the two groups. Of note, some women had more than one risk factor for spontaneous preterm birth. A summary of maternal demographics and risk factors for spontaneous preterm birth can be seen in Table 1.

**Table 1:** Maternal Demographics and Risk Factors for Spontaneous Preterm Birth

	Preterm (*n* = 13)	Term (*n* = 40)	*P* value
Mean age, years (range)	34(23-43)	34 (24-45)	0.953
Mean body mass index, kg/m^2^(range)	29(19-39)	28 (19-37)	0.769
Mean body mass index, kg/m^2^ (range)	29(19–39)	28 (19–37)	0.769
Ethnicity			
- Asian	2 (15%)	2 (5%)	0.311
- Black	5 (38%)	17 (43%)	
- White	6 (46%)	15 (38%)	
- Unrecorded	0 (0%)	6 (15%)	
Parity			
- 0	7 (54%)	11 (28%)	0.103
- 1	1 (8%)	17 (43%)	
- 2	1 (8%)	5 (13%)	
- 3	1 (8%)	4 (10%)	
- > 4	3 (23%)	3 (8%)	
Risk factors for sPTB			
- Previous sPTB	6 (46%)	10 (40%)	0.075
- Previous mid-trimester loss	5 (38%)	20 (50%)	0.469
- Previous PPROM	1 (8%)	11 (28%)	0.138
- Previous cervical surgery	4 (31%)	5 (13%)	0.127
- Previous FDCS	0 (0%)	3 (8%)	0.309
- Uterine anomaly	1 (8%)	1 (3%)	0.393

Maternal demographics and risk factors for spontaneous preterm birth in the study population. *sPTB* = spontaneous preterm birth, *PPROM* = preterm pre-labour rupture of membranes, *FDCS* = full dilatation caesarean section

Of the 40 women who delivered term 53% (*n* = 21) had a history-indicated TVC, 45% (*n* = 18) had an ultrasound indicated TVC and 2% (*n* = 1) had a rescue TVC. Within the term group 85% (*n* = 34) had a Mcdonald cerclage and 15% (*n* = 6) had a Shirodkar cerclage. The mean gestational age at TVC insertion in the term group was 18 + 3 weeks (range 10 + 5 to 23 + 5) and the mean interval to scan following TVC insertion was 16 days (range 2–63). There was no significant difference in these parameters between term and preterm groups. See Table 2 for cerclage indication, subtype and interval to scan details. Table 2: Indications for transvaginal cerclage, subtype of transvaginal cerclage and scan findings. TVC = transvaginal cerclage. *Pre-cerclage findings were missing from 7 cases (history-indicated cerclages).

**Table 2:** Cerclage Indication, Subtype and Scan Findings

	Preterm (*n* = 13)	Term (*n *= 40)	*P* value
Indication			
- Indication	5 (38%)	21 (53%)	0.375
- Ultrasound	6 (46%)	18 (45%)	0.942
- ‘Emergency’/rescue	2 (15%)	1 (2%)	0.081
Mean gestational age at cerclage insertion, weeks + days (range)	18 + 3 (range 8 + 3–23 + 3)	16 + 3 (range 10 + 5–23 + 5)	0.180
Cerclage subtype			
- Mcdonald	11 (85%)	34 (85%)	0.973
- Shirodkar	2 (15%)	6 (15%)	0.973
Mean days from cerclage insertion to scan (range)	15 (6–42)	16 (2–63)	0.184
Mean cervical length before cerclage insertion*, mm (range)	17 (7–27)	26 (3–44)	0.009
Additional ultrasound features pre-cerclage*			
- Funnelling	6 (46%)	8 (21%)	0.084
- Amniotic fluid sludge	3 (23%)	5 (14%)	0.403
Mean cervical length after cerclage insertion, mm (range)	26 (10–40)	30 (15–48)	0.190
Mean length above the cerclage (distance from cerclage to internal os), mm (range)	11 (4–20)	16 (4–30)	0.008
Mean height of cerclage (distance from external os to cerclage) mm (range)	15 (6–27)	14 (5–32)	0.573
Mean relative position of cerclage	0.41 (0.20–0.61)	0.52 (0.20–0.86)	0.024
Additional ultrasound features post-cerclage			
- Funnelling	5 (38%)	3 (8%)	0.007
- Amniotic fluid sludge	3 (23%)	0 (0%)	0.002

Indications for transvaginal cerclage, subtype of transvaginal cerclage and scan findings. *TVC* = transvaginal cerclage. *Pre-cerclage findings were missing from 7 cases (history-indicated cerclages)

There was a difference in the mean cervical length before the TVC insertion in those who delivered preterm versus term (mean difference − 8.95 mm, 95% CI -15.58 to -2.42, *p* = 0.009) but not the cervical length after TVC insertion (mean difference − 3.28 mm 95% CI − 8.24 to 1.67, *p* = 0.190).

There was a significant difference in the relative position of the TVC. The mean proportion of length above the TVC and the total cervical length was 0.41 in those who delivered preterm compared to 0.52 in those who delivered term; mean difference 0.11 (95% CI 0.016–0.21; *p* = 0.024).

There was a significant difference between the length above the TVC, distance from the TVC to the internal os, between the two groups (mean difference 5.42 mm, 95% CI 1.47 to 9.37, *p* = 0.008). Those who delivered preterm had a shorter length above the cerclage than those who delivered term (mean 11 mm [range 4–20] versus 15 mm [range 4–30]; *p* = 0.008). There was no significant difference in the TVC height (distance from the external os to the TVC), between the two groups (mean difference 1.13 mm, 95% CI − 2.87 to 5.14, *p* = 0.573).

**Fig. 3:**
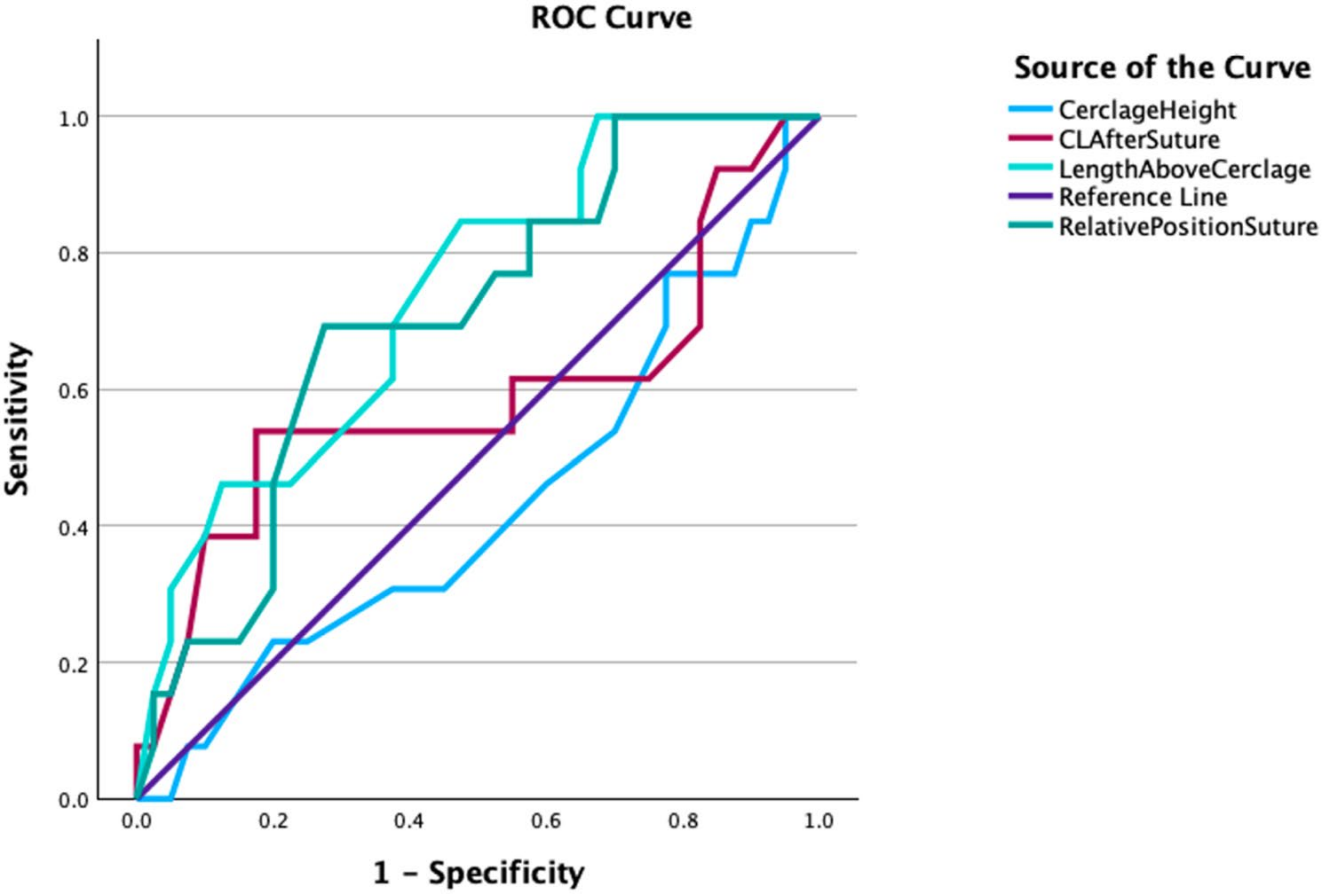
ROC curve for cerclage measurements

When assessing the ROC curve for these measurements in the prediction of spontaneous preterm birth, length above the TVC and relative position of TVC had good prediction (AUC 0.73 and 0.74), while cervical length and cerclage height had no relationship (AUC 0.43 and 0.59 respectively), see Table 3; Fig. 3.

**Table 3:** Area Under the ROC Curve for Post-Cerclage Ultrasound Parameters

	Area	Standard Error	Asymptotic Significance	95% Confidence Interval
Cervical length after TVC	0.587	0.106	0.416	0.378-0.795
Length above the cerclage	0.736	0.076	0.002	0.587–0.885
Relative position of cerclage	0.704	0.079	0.010	0.548–0.859
Cerclage height	0.431	0.095	0.465	0.245–0.616

Area under the ROC curve for ultrasound parameters. *ROC* = receiver operator characteristic; *TVC* = transvaginal cerclage

Using the ROC curve, an optimum threshold for relative position of TVC of 0.425 was identified. When applying this threshold to the data set, the sensitivity was 69% (9/13) and the specificity was 75% (30/40), with a positive predictive value of 47% (9/19) and the negative predictive value of 88% (30/34). This indicates that when the relative position was below the threshold, the measure performed well in correctly identifying true negatives, with a high negative predictive value suggesting that cases classified as low risk were unlikely to be affected. However, values exceeding the threshold were less reliable for confirming the condition, as reflected by the lower positive predictive value.

Calibration assessment demonstrated good agreement between predicted and observed risks at lower probability ranges, with slight underestimation in the mid-range and greater variability at higher predicted probabilities, likely reflecting the small sample size. The Hosmer–Lemeshow test did not indicate lack of fit (Chi square² = 10, *p* = 0.214), supporting adequate overall calibration.

Those who delivered preterm were more likely have a funnel (chi-square = 7.34, *p* = 0.007) and amniotic fluid sludge present (chi-square = 9.79, *p* = 0.002) on ultrasound after TVC; there was no difference between the groups in the findings of funnel (chi- square 4.95, *p* = 0.084) or amniotic fluid sludge (chi-square 1.82, *p* = 0.403) before the TVC. See Table 2 for a summary of scan findings before and after TVC placement.

## Discussion

This study suggests that relative position of TVC is lower in those who subsequently delivered preterm in comparison to those that subsequently delivered at term (*p* = 0.024). A shorter cervical length above the TVC is associated with an increased risk of subsequent preterm delivery (*p* = 0.008). The presence of funnelling and amniotic fluid sludge after cerclage is also associated with an increased risk of subsequent preterm delivery (*p* = 0.007 and *p* = 0.002 respectively). To our knowledge this is the first study to evaluate TVC in this way.

We postulate that a shortening cervical length above the TVC, as detected on the first ultrasound following TVC insertion, may reflect active change in the cervix which may proceed to further cervical shortening and dilatation. This is also illustrated by the relative position of TVC. For example, a cerclage measured to be 20 mm from the external os may be considered ‘high’. However, if the overall cervical length is 30, then there would be 10 mm of closed cervical length above the cerclage and if the cervical length was 25 mm, there would only be 5 mm of closed cervical length above the cerclage. Here relative position may be more helpful, in this case 0.30 versus 0.20. Indeed, when assessing the ROC curves for these parameters, length above the TVC and relative position of TVC had good prediction for spontaneous preterm birth (AUC 0.73 and 0.74), while cervical length and cerclage height had no relationship (AUC 0.43 and 0.59 respectively). Moreover, relative position showed adequate overall calibration.

Other studies suggest that there is an association between cerclage height and preterm delivery, our findings may differ as we analysed all types of TVC together due to the small numbers in each group. The current evidence for TVC height as a predictor for spontaneous preterm birth is conflicting depending on the type of TVC investigated, with some studies suggesting that it is a good predictor in history and emergency cerclage [10–12]and other studies suggesting it’s benefit in ultrasound indicated cerclage [9]. The difference between these studies may be related to sample sizes. Moreover, previous studies investigating cervical length after cerclage, were able to assess change in cervical length after cerclage insertion by taking longitudinal measurements [7], a key difference. Even if there is a relationship between cervical length after cerclage and spontaneous preterm birth, the relative position is likely to be better.

Another key finding in this study was the association between post-cerclage cervical funnelling and amniotic fluid sludge after cerclage and spontaneous preterm birth (*p* = 0.007 and *p* = 0.002 respectively). Again, these findings may reflect dynamic change above the cerclage, which may occur before shortening of the cervix. Post-cerclage funnelling was more commonly persistent (6/8) than new (2/8); resolution of pre-cerclage funnelling was documented in 7 cases. Similarly, findings of amniotic fluid sludge were more frequently persistent (2/3) than new (1/3) and had resolved in in 6 cases.

These findings are in keeping with previous studies which demonstrated that women with a history-indicated or emergency TVC (indicated by vaginal examination), were over 5 times more likely to deliver preterm if they had evidence of funnelling on a subsequent ultrasound [15]. Other studies also demonstrate that funnelling after a history indicated TVC is associated with preterm delivery [16, 17] .

Amniotic fluid sludge, or ‘the presence of dense aggregates of particulate matter in the proximity of the cervical os’, is thought to reflect intra-amniotic inflammation or infection [14, 18]. The presence of amniotic fluid sludge has been associated with preterm delivery in asymptomatic women at high risk of spontaneous preterm birth and in those with a short cervical length [14, 18]. However, a previous retrospective study assessed amniotic fluid sludge in those with a history or ultrasound indicated TVC and did not find a difference in the rate of preterm delivery or mean gestational age of delivery between those with amniotic fluid sludge and those without [13]. A potential reason for this difference in findings is that this study also included emergency cerclages. Future work, ideally prospectively collected and with larger numbers, should be done to explore this further.

There were several limitations to this study. The sample size was small (although one of the largest of its kind) and it was retrospective in nature. In addition, all types of TVC - history-indicated, USS-indicated and emergency - were analysed together. The small numbers within each cerclage type, did not allow for meaningful sub-analyses; however, both the indication for cerclage and the type of cerclage are likely to influence post-cerclage ultrasound findings. Moreover, there was significant variation in the time between cerclage insertion and subsequent ultrasound examination, from 2 to 63 days. Other factors, which may influence outcomes, including cerclage tension and operator were not evaluated.

Despite these limitations, this study uses a novel approach to support the evidence that ultrasound assessment of the cervix following a TVC is useful in predicting those likely to deliver preterm following intervention. At present guidelines do not recommend routine post TVC ultrasound and practice varies between obstetric units [3, 19]. However, to better facilitate preterm prediction and optimization of the preterm neonate – i.e. by the administration of timely corticosteroids, admission to a gestation appropriate obstetric and neonatal unit and magnesium sulphate administration for fetal neuroprotection – following intervention this practice should be reviewed.

Moreover, the exact ultrasound parameters - and combination of parameters – that most accurately predict preterm in the context of TVC remain to be fully elucidated. Large prospective studies are needed to assess this in further detail, including analyses across different preterm gestational age groups, assessment of TVC sub-types and consideration of specific spontaneous preterm birth risk factors. Further work exploring the role of longitudinal measurements of post-TVC USS parameters – particularly the including relative position and length above the cerclage - would also be valuable. In addition, complementary biomechanical assessments, such as cervical stiffness measured via cervical elastography or aspiration-based devices, are an emerging area of investigation and may offer additional insight when evaluating the cervix after cerclage [20, 21].

## Conclusion

Ultrasound assessment of the cervix following TVC placement is likely to be beneficial in determining which patients are at risk of preterm delivery despite intervention. The relative position of the TVC is a good predictor of spontaneous preterm birth after cerclage and is likely better than cervical length and height of cerclage. The length above the TVC, and presence of a funnel and amniotic fluid sludge are also factors associated with subsequent preterm delivery. They may suggest dynamic changes occurring above the level of the TVC. Although a TVC which is placed high in the cervix, has historically been associated with prolongation of pregnancy and term delivery, other parameters may add to the clinical picture to best guide ongoing management. This was a small retrospective study, and so larger prospective studies are required to further investigate this.

## Data Availability

The data that support the findings of this study are not openly available due to reasons of sensitivity and are available from the corresponding author upon reasonable request.

## Abbreviations

AUC: area under the curve
ROC: receiving operarting characteristic
sPTB: spontaneous preterm birth
TVC: transvaginal cerclage
